# A Novel *flaB* Gene-Based Profiling Approach for the Rapid and Accurate Detection of *Borreliella* and *Borrelia* Species in Ticks

**DOI:** 10.3390/pathogens14050506

**Published:** 2025-05-21

**Authors:** Abigail Dorothea Taylor, Artur Trzebny, Małgorzata Łośko, Jerzy Franciszek Michalik, Miroslawa Dabert

**Affiliations:** 1 Molecular Biology Techniques Laboratory, Faculty of Biology, Adam Mickiewicz University, 61-614 Poznan, Poland; abigail.taylor@amu.edu.pl (A.D.T.); arturtrzebny@amu.edu.pl (A.T.); malgorzata.losko@amu.edu.pl (M.Ł.); 2 Department of Animal Morphology, Faculty of Biology, Adam Mickiewicz University, 61-614 Poznan, Poland; jerzy.michalik@amu.edu.pl

**Keywords:** Borreliaceae, molecular screening, flagellin, *Borrelia burgdorferi* sensu lato, targeted amplicon sequencing, 16S profiling

## Abstract

The increasing incidence of tick-borne diseases in Europe necessitates the development of accurate and high-throughput molecular tools for detecting pathogens in tick populations. In this study, we present a novel *flaB* gene-based profiling method for the detection and identification of *Borrelia* and *Borreliella* species in *Ixodes ricinus* ticks, combining newly designed primers with next-generation sequencing (NGS). The method was evaluated alongside conventional nested PCR targeting the *flaB* gene, as well as microbial profiling based on the V4 region of the *rrs* gene, using tick DNA extracted from 1088 specimens pooled into 94 samples. Our results demonstrate that the *flaB* gene-based profiling approach was the highest-performing out of the three methods, detecting Borreliaceae DNA in 83 DNA pools, compared to 58 and 56 pools using nested PCR and V4 *rrs* profiling, respectively. A total of 23 distinct *flaB* sequence variants were identified, corresponding to five Borreliaceae species: *Borreliella afzelii*, *Bl. garinii*, *Bl. valaisiana*, *Bl. burgdorferi*, and *Borrelia miyamotoi*. Additionally, the method enabled putative strain-level discrimination within species. Our results highlight the value of *flaB* gene-based profiling as a robust tool for ecological and epidemiological studies of Borreliaceae diversity in ticks.

## 1. Introduction

The observed increase in the incidence of tick-borne diseases (TBDs) worldwide is a challenge for health professionals, who should also be involved in raising public awareness. This is particularly important in Europe, where the risk of diseases transmitted by *Ixodes ricinus* ticks is growing, including in Poland [1]. This is a growing concern due to the observed expansion of *I. ricinus* abundance and the spread of populations of this tick in Europe [2]. There is, therefore, an urgent need to develop both more accurate diagnostic techniques and advanced methods for researchers to study and monitor tick-borne pathogens, as well as to introduce more effective strategies to prevent TBDs [3,4,5].

The most common TBD in the northern hemisphere is Lyme borreliosis (LB), which is caused by spirochetes of the Borreliaceae family [6,7]. Molecular phylogenetic analyses have shown that the members of this family can be grouped into two clades, and that the pathogenic species within each clade can be distinguished by different disease symptoms and by their specific relationships with the tick vector and reservoir hosts [8]. On the basis of these data, two genera have been proposed: the genus *Borreliella*, formerly known as the *Borrelia burgdorferi* sensu lato complex, which includes the three most important LB agents in Europe, i.e., *Bl. afzelii*, *Bl. garinii*, and *Bl. burgdorferi*; and the genus *Borrelia*, comprising the spirochetes that cause tick-borne relapsing fever (TBRF), including *Borrelia miyamotoi*, the etiological agent of Borrelia miyamotoi disease. *Borrelia miyamotoi*, transmitted by *I. ricinus*, is the only species causing TBRF found in Europe [8,9]. Considering the extensive diversity of Borreliaceae species and their varying zoonotic potential, it is crucial to conduct systematic and continuous monitoring of the occurrence and distribution of *Borrelia* and *Borreliella* species in the environment.

Various methods are used for Borreliaceae DNA detection and species identification, including serological surveys, environmental DNA sampling, host tissue analysis, and molecular assays [10,11,12,13]. Molecular techniques based on the polymerase chain reaction (PCR) are among the most widely applied methodologies, due to their fast processing time and high analytical sensitivity. The application of group- or species-specific primer sets, followed by PCR amplification of a target gene fragment and subsequent sequencing of the amplicons, enables accurate and reproducible identification of the taxon of interest.

Commonly used DNA markers for *Borreliella* species detection include genes encoding the small subunit ribosomal RNA gene (16S rRNA, hereafter *rrs*), outer-surface proteins (e.g., *ospA*, *ospB*, *ospC*), and flagellin protein (*flaB*). However, the genes encoding outer-surface proteins (Osps) do not tend to have homologs between the genera; instead, *Borrelia* spirochaetes utilize variable membrane proteins (Vmps) in the place of Osps [14,15]. Furthermore, vmp-like sequences of *Borreliella* spirochaetes show too-high sequence variability for precise *Borreliella* species assignment [16]. A screening assay based on the hypervariable V2 region of the *rrs* has also been proposed, which enables the targeted amplification of Borreliaceae DNA [17]. Additional universal DNA markers that allow for the simultaneous detection of both *Borrelia* and *Borreliella* species include *rrs* gene-based microbial profiling targeting the V3 and/or V4 *rrs* regions, which can be applied across all bacteria due to the use universal primers, and the *flaB* gene, which is present in all members of the Borreliaceae family. The *flaB* gene exhibits conserved nucleotide substitutions among spirochaete species, facilitating the design of specific PCR primers, yet maintains sufficient sequence variability to enable differentiation at the species level [18,19,20].

Most methods currently used for *flaB* gene analysis are based on a nested PCR approach and sequencing of the second-round PCR product [17,21,22,23,24]. However, this standard approach based on Sanger sequencing of PCR amplicons may result in false-negative detections in co-infected samples, particularly when one spirochaete species dominates in abundance over other co-existing taxa, a phenomenon previously reported for other pathogenic microorganisms [25]. An effective solution to this limitation may be provided by high-throughput assays based on microfluidic real-time PCR, utilizing digital PCR (dPCR) technology. This method enables the simultaneous detection of dozens of tick-borne pathogens (TBPs) [26]. Nevertheless, this approach still relies on highly specific primers and probes, which may result in failure to detect strains that exhibit sequence variation within the targeted gene regions. In contrast, methods developed in recent years for the taxonomic profiling of microbial communities resolve this problem, because they are based on parallel sequencing of all amplicons in the sample using a next-generation sequencing (NGS) approach. Such approaches provide information not only on the amplicon sequence variants (ASVs) in the sample, but also on their relative abundance, which may reflect the relative contribution of a given taxon to the microbial community. By using degenerate primer sets optimized to detect a broad spectrum of taxa, this strategy may serve as an alternative to the recently developed multiplex PCR amplicon sequencing (MPAS) approach [27], which enables the identification of *Borrelia* and *Borreliella* species, including co-infections [20].

Therefore, the aim of this study was to develop a novel DNA-based profiling approach for the rapid detection and species-level identification of Borreliaceae members based on the *flaB* marker. To achieve this, we designed a new group-specific primer set for the PCR amplification of a short *flaB* gene fragment, enabling the detection of both *Borrelia* and *Borreliella* species. The performance of the primers was evaluated using DNA extracted from *I. ricinus* ticks, the primary vector of spirochetes in Europe. The results of *flaB* gene-based profiling were compared with those obtained using a conventional nested PCR assay targeting the *flaB* gene, as well as with taxonomic profiles of tick bacterial communities based on the V4 region of the *rrs* gene (V4 *rrs*).

## 2. Materials and Methods

### 2.1. Tick Collection, DNA Extraction, and Pooling of Isolates

Questing *I. ricinus* ticks were collected in May and June over a period of 11 years, from 2012 to 2022, in a mixed forest stand at the Rusalka Lake in the city of Poznan, west-central Poland (N 52.426389, E 16.877778), using a flag-dragging method, and stored in 96% ethanol until analyses. Ticks were assigned to species by examination of morphological features according to a key by Siuda (1993) [28]. In total, 1088 *I. ricinus* ticks were collected, including 170 females and 918 nymphs; from this group, up to 20 females and 100 nymphs were selected per year for screening for Borreliaceae DNA.

Total DNA was extracted from ticks using the ammonium hydroxide-based method described by Rijpkema et al. (1996) [29]. In total, 94 DNA pools were prepared by combining 5 µL of each individual DNA extract. Before pooling, DNA extracts were checked for the presence tick DNA by conventional PCR amplification of the cytochrome *c* oxidase subunit I gene fragment (COI) using bcdF01 and bcdR04 primers, as described previously [30]. This resulted in 54 pools containing DNA from 12 ticks each, and 40 pools containing DNA from 11 ticks each; adults were randomly pooled with nymphs. The positive control was a DNA extract from a tick positive for *Bl. valaisiana*, collected for a previous study [31]. Positive and negative controls, including blank DNA extraction controls and no-template PCR controls, were processed, amplified, and sequenced alongside the test samples.

### 2.2. Designing PCR Primers for flaB Gene-Based Profiling

A new Borreliaceae-specific primer set for PCR amplification of a *flaB* gene fragment suitable for NGS was developed based on sequence data published in GenBank [https://www.ncbi.nlm.nih.gov/ (accessed on 15 April 2024)]. In total, 22 *flaB* sequences representing *Bl. afzelii* (JANAGAF1173 and PKo strains), *Bl. americana* (SCW41), *Bl. bavariensis* (NT24 and PBi), *Bl. bissettiae* (PGeb), *Bl. burgdorferi* (Bol26), *Bl. chilensis* (VA1), *Bl. finlandensis* (Z11), *Bl. garinii* (CIP 103,362 and JAASAAF1041), *Bl. japonica* (Miyazaki 2E), *Bl. kurtenbachii* (25015), *Bl. lusitaniae* (PotiB2), *Bl. mayonii* (MN14-1420 and MN14-1539), *Bl. sinica* (CMN3), *Bl. spielmanii* (PMew), *Bl. yangtzensis* (CW61 and CW62), and *B. miyamotoi* (HT31 and NL-IR-1) (Table S1) were aligned using BioEdit 7.2.5. [32]. Initial primers were analyzed in Oligo Analyzer version 3.1 (Integrated DNA Technologies Inc., Coralville, IA, USA) to check the difference in melting temperatures and to search for possible primer secondary structures. The final abi-flaB primer set, comprising the degenerate primers abi-flaB forward (WGCWTCTGATGATGCTGCTG) and abi-flaB reverse (TATTGVGCYTGATCAGCAATTC), was designed to amplify a 276 bp fragment of the *flaB* gene, corresponding to amino acid positions Met46 to Asn122 in the flagellin protein sequence (e.g., *Bl. afzelii* strain JANAGAF1173, NZ_CP075209.1:c148380-147370).

### 2.3. Screening for Borreliaceae DNA Using Nested PCR Test

An approximately 600 bp fragment of the *flaB* gene, commonly used for the conventional detection of Borreliaceae DNA, was amplified using a nested PCR approach. The outer PCR was performed with primers 132f (TGGTATGGGAGTTTCTGG) and 905r (TCTGTCATTGTAGCATCTTT) [16]. The reaction was carried out in a final volume of 6 µL, containing 1 × Type-it Microsatellite PCR Master Mix (Qiagen, Hilden, Germany), 0.25 µM of each primer, and 1 µL of DNA template. The thermal cycling conditions were as follows: initial denaturation at 95 °C for 5 min, followed by 40 cycles of denaturation at 95 °C for 30 s, annealing at 50 °C for 45 s, and extension at 72 °C for 1 min, with a final extension step at 72 °C for 7 min. The outer PCR product was subsequently diluted 1:10 with nuclease-free water (Milli-Q) and used as a template for the inner PCR. The inner PCR amplification was carried out with the use of 220f (CAGACAACAGAGGGAAAT) and 823r (TCAAGTCTATTTTGGAAAGCACC) primers [16]. The reaction was performed in a final volume of 10 µL, containing 1 × Type-it Microsatellite PCR Master Mix, 0.2 µM of each primer, and 1 µL of DNA template. The thermal cycling conditions were the same as for the first-round reaction. After completion of the PCR, 2.5 µL of the reaction mixture was separated on 1% agarose gel and visualized using GelRed Nucleic Acid Gel Stain (Biotium, Inc., Fremont, CA, USA) to check the amplification efficiency.

### 2.4. Screening for Borreliaceae DNA Using V4 rrs Gene-Based Profiling

The V4 hypervariable region (about 250 bp) of the bacterial *rrs* gene was amplified using primers V4F (GATCAGCAGCCGCGGTAATA) [33] and V4R (GGACTACCAGGGTATCTAA) [34], both fused at their 5′ ends with dual-indexed Ion Torrent adapters. PCRs were carried out in a final volume of 10 µL, containing 1 × HOT FIREPol polymerase (Solis BioDyne, Tartu, Estonia), 0.2 µM of each fusion primer, and 1 µL of DNA template. Each reaction was performed in technical duplicate, and the two replicates were pooled after amplification. The thermal cycling conditions were as follows: initial denaturation at 95 °C for 12 min, followed by 30 cycles of denaturation at 95 °C for 15 s, annealing at 50 °C for 90 s, and extension at 72 °C for 30 s, with a final extension step at 72 °C for 5 min. Following PCR, technical replicates were combined, and 2.5 µL of each product was electrophoresed on a 1% agarose gel to assess amplification efficiency. All PCR products were subsequently combined to create a single V4 *rrs* amplicon library.

### 2.5. Screening for Borreliaceae DNA Using flaB Gene-Based Profiling

A 276 bp fragment of the *flaB* gene was amplified using abi-flaB forward and reverse primers (see Section 2.2), fused at their 5′ ends with dual-indexed Ion Torrent adapters. PCRs were carried out as described for V4 *rrs* gene-based profiling. The thermal cycling conditions were as follows: initial denaturation at 95 °C for 12 min, followed by 40 cycles of denaturation at 95 °C for 15 s, annealing at 55 °C for 30 s, and extension at 72 °C for 30 s, with a final extension step at 72 °C for 5 min. All PCR products were subsequently combined to create a single *flaB* amplicon library.

### 2.6. Library Preparation and Sequencing

The V4 *rrs* and *flaB* amplicon libraries were individually purified using a 2% E-Gel SizeSelect II Agarose Gel System (Invitrogen, Carlsbad, CA, USA), following the manufacturer’s protocol. Library concentration and fragment size distribution were assessed using a High-Sensitivity D1000 ScreenTape assay on a 2200 TapeStation system (Agilent Technologies, Santa Clara, CA, USA). Clonal template amplification was carried out with the Ion Torrent OneTouch System II and the Ion Torrent OT2 Kit, according to the manufacturer’s instructions. Sequencing was performed on an Ion 540 chip using the Ion 540 Kit-OT2 and Ion S5 system (Life Technologies, Carlsbad, CA, USA), following the manufacturer’s guidelines.

### 2.7. Bioinformatic and Statistical Analyses

Amplicon sequencing was performed using the Ion Torrent S5 system (Life Technologies, Carlsbad, CA, USA), according to the manufacturer’s instructions, to yield approximately 50,000 and 250,000 reads per sample of the *flaB* and V4 *rrs* library, respectively. Bioinformatic analysis was conducted using FASTQ data and a custom workflow. Briefly, sequence reads shorter than 200 bp were removed from the dataset using Geneious Prime 2023.1.2 (Biomatters Ltd., Auckland, New Zealand). Quality filtering was performed with the FastX-Toolkit, retaining sequences with at least 50% of bases having a quality score of ≥25 [35]. The quality-filtered sequences were then demultiplexed into individual index combinations in Geneious Prime. Subsequently, sequences were trimmed at both the 5′ and 3′ ends to remove PCR primer sequences in Geneious Prime using default settings, with the maximum number of mismatches increased to three. Denoising was performed using the DADA2 denoise-pyro method implemented in QIIME2 version 2024.10 [36,37] and feature-classifier, feature-table, metadata, quality-filter, stats, taxa, demux, and dada2 plugins to generate amplicon sequencing variants (ASVs). Chimeric sequences were identified within individual samples using the dada2 plugin, and those consistently flagged as chimeric across a substantial fraction of samples were removed from the dataset. ASVs detected in control samples were excluded from the dataset using the UNCROSS2 algorithm [38].

ASVs obtained from the V4 region of the *rrs* gene were taxonomically assigned by comparison against the SILVA SSU rRNA database (version 138.1) [www.arb-silva.de (accessed on 15 September 2024)] [39,40,41], while ASVs obtained from the *flaB* gene fragment of Borreliaceae were compared against the GenBank database using the blastn tool [42], with the megablast algorithm optimized for highly similar sequences [43]. The blastn search was restricted to records derived from type material, and excluded uncultured and environmental sample sequences, with a 97% identity threshold applied for taxon assignment. Finally, ASVs of the *flaB* gene fragment were translated into amino acid sequences using bacterial codons in GeneDoc 2.7 [44].

Pairwise comparisons between Borreliaceae DNA detection methods were performed using McNemar’s exact test for paired binary data [45], conducted in R version 4.5.0 via RStudio version 2024.12.1, with the ‘readr’ package version 2.1.5 and the ‘dplyr’ package version 1.1.4, and the exact 2 × 2 package version 1.6.9.

### 2.8. Phylogenetic Analysis

A total of 55 *flaB* sequences, including 23 sequence variants found in this study, sequences representing *Borreliella* (20) and *Borrelia* (11) species, and uncultured Spirochaetia bacterium to root the tree, were used for phylogenetic analysis (Table S2). Sequences were aligned manually in GeneDoc 2.7. The final alignment of 204 nucleotide positions was used to construct a phylogenetic tree using FastTree 2.1.11 and a GTR + G model, as implemented in Geneious Prime 2024.0.7 (GraphPad Software LLC d.b.a Geneious, Boston, MA, USA). Statistical support for branches was estimated by the Shimodaira–Hasegawa test (SH) [46]. The tree was edited in MEGA 7.0.26 [47] and Corel Draw v. X5.

## 3. Results

### 3.1. Screening by Conventional Nested PCR

**Figure 1:**
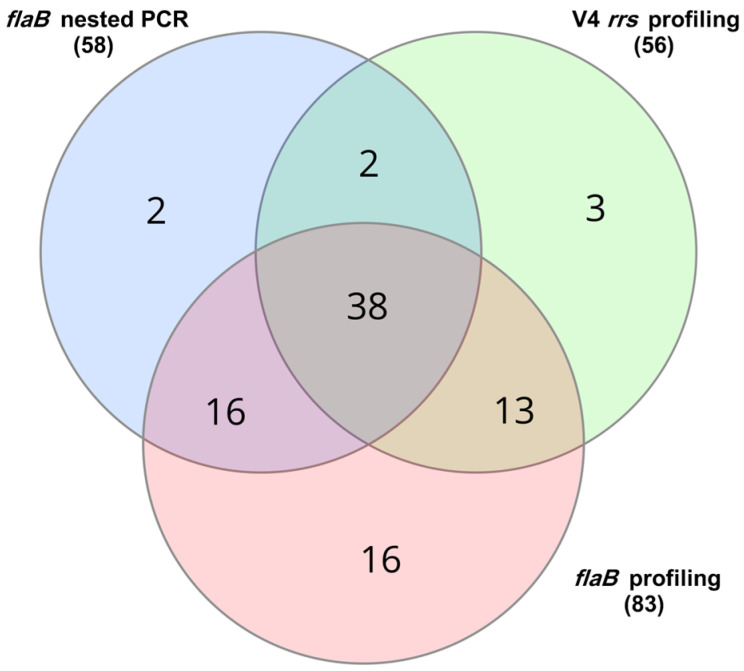
A Venn diagram illustrating the number of DNA pools positive for Borreliaceae DNA, as determined by three molecular approaches: nested PCR targeting the *flaB* gene (blue), V4 *rrs* gene profiling (green), and *flaB* gene profiling (pink). The total number of positive pools detected by each method was 58 for *flaB* nested PCR, 56 for V4 *rrs* gene-based profiling, and 83 for *flaB* gene-based profiling. Overlapping areas indicate the number of DNA pools identified as positive by two or all three methods.

The nested PCR test based on the amplification of an approximately 600 bp fragment of the *flaB* gene unequivocally confirmed the presence of Borreliaceae DNA in 58 out of 94 DNA samples, which had been prepared by pooling DNA extracts obtained from a total of 1088 individual ticks (Figure 1 and Figure S1). Amplicons generated by the nested PCR approach were not subjected to Sanger sequencing, as each DNA pool contained more than 10 individual DNA isolates derived from different ticks. Consequently, it was assumed that the PCR products could be heterogeneous in composition, potentially containing multiple flaB gene variants, which would complicate or preclude accurate interpretation of the Sanger sequencing results.

### 3.2. Screening by V4 rrs Gene-Based Profiling

Amplification and sequencing of the V4 region of the *rrs* gene confirmed the presence of Borreliaceae DNA in 56 DNA pools. However, only 40 of these Borreliaceae-positive pools were consistently detected by both the *flaB* nested PCR assay and the V4 *rrs* sequencing approach (Figure 1, Table S3).

### 3.3. Screening by flaB Gene-Based Profiling

We identified seven DNA pools that were not detected using *flaB* gene-based profiling, including two that were detected by both the nested PCR and the V4 rrs marker (Figure 1, Table S3). Nevertheless, screening by *flaB* gene-based profiling demonstrated the highest performance among the three approaches tested, detecting Borreliaceae DNA in 83 out of 94 tick DNA pools. Notably, this included 29 DNA pools that had tested positive in only one of the previous methods—either the *flaB* nested PCR assay or the V4 *rrs* gene-based profiling.

To assess the differences in detection performance among the three molecular methods, pairwise comparisons were performed using McNemar’s exact test. The results revealed statistically significant differences in detection frequency between the *flaB* gene-based profiling approach and both conventional nested PCR (*p* = 1.09 × 10^−5^) and V4 *rrs* profiling (*p* = 7.43 × 10^−6^). In contrast, no significant difference was observed between the nested PCR and V4 *rrs* profiling methods (*p* = 0.8642). These results confirm that the *flaB* gene-based profiling is significantly more effective in detecting Borreliaceae DNA in pooled tick samples compared to the other two approaches (Table 1).

### 3.4. Flagellin Gene Sequence Variants

Using *flaB* gene-based profiling, a total of 23 *flaB* sequence variants were identified in the *I. ricinus* DNA analyzed in this study (Table S2; GenBank accession numbers PQ727213–PQ727235; note that sequence variant 3 was excluded from this dataset). Blastn searches against the GenBank database revealed identical reference sequences for fewer than half of the detected variants (11 out of 23).

**Figure 2:**
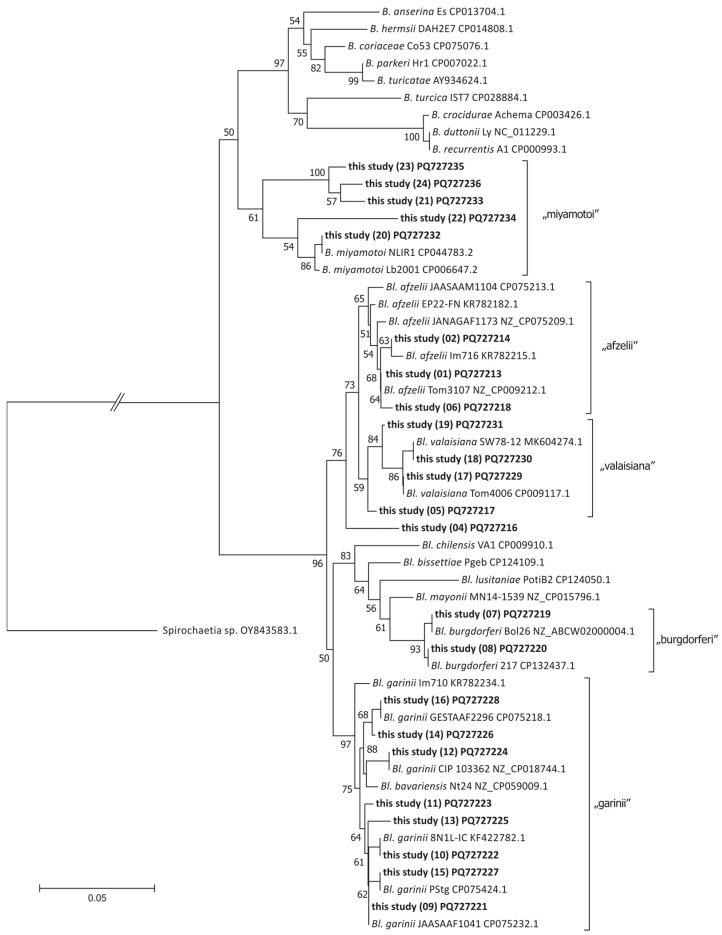
Phylogenetic tree generated using FastTree to assign *flaB* DNA sequence variants obtained through *flaB* gene profiling (labeled ‘this study’) to Borreliaceae species. Branch support values were calculated using Shimodaira–Hasegawa (SH) test; only support values > 50% are shown.

Phylogenetic analysis (Figure 2) enabled unambiguous assignment of the *flaB* sequence variants to five Borreliaceae species: *Bl. afzelii* (variants 1, 2, 6, 19), *Bl. valaisiana* (variants 17, 18), *Bl. burgdorferi* (variants 7, 8), *Bl. garinii* (variants 9–16), and *B. miyamotoi* (variants 20–24). One sequence variant (variant 4; PQ727216) was positioned, in our phylogenetic analysis, as a sister lineage to the clades containing *flaB* sequences of *Bl. afzelii* and *Bl. valaisiana*. However, blastn comparison indicated the highest sequence similarity to *Bl. afzelii*, sharing 196 out of 202 nucleotides (97% identity) with the *Bl. afzelii* clone EP-22-FN (GenBank accession no. KR782182.1). Consequently, variant 4 was assigned to *Borreliella* sp.

All *flaB* sequence variants were translated into amino acid sequences, resulting in a 67-amino-acid fragment without stop codons (Figure S2). In total, three different amino acid sequence variants were obtained: one corresponding to *B. miyamotoi*, and two others—differing by a single amino acid position—found within the genus *Borreliella*. One of these was shared by *Bl. afzelii* and *Bl. valaisiana* (ASV01-06, ASV17-19), while the other was common to *Bl. garinii* and *Bl. burgdorferi* (ASV07-16).

### 3.5. Borreliaceae Species Identified Using flaB Gene-Based Profiling

The most frequently identified species based on *flaB* gene-based profiling was *Bl. afzelii*, detected in 51 out of 83 Borreliaceae-positive DNA pools (61.45%; 95% CI: 50.69–71.19%). This was followed by *Bl. valaisiana* (37/83; 44.58%; 95% CI: 33.86–55.30%), *Bl. garinii* (28/83; 33.73%; 95% CI: 23.60–43.86%), *Bl. burgdorferi* (10/83; 12.05%; 95% CI: 6.68–20.78%), and *B. miyamotoi* (21/83; 25.30%; 95% CI: 15.95–34.65%).

### 3.6. Microbial Profiling Using V4 rrs Marker

Microbial profiling based on the V4 *rrs* marker revealed 13 distinct sequence variants (GenBank accession numbers PV151552–PV151564) across 56 DNA pools. Notably, despite the considerable variability observed in the *flaB* gene sequences of *B. miyamotoi*, all DNA pools positive for this species shared the same V4 *rrs* sequence variant (GenBank accession no. PV151554).

In comparison to earlier research conducted on the same area [31], two novel V4 *rrs* sequence variants were identified in this study: ASV20, which exhibited 99% identity to the *Bl. lusitaniae* strain Poti B2 sequence, and ASV21, which was identical to sequences reported for *Bl. americana* and *Bl. mayonii*.

The most frequently detected V4 *rrs* sequence variant, ASV06, was present in 24 DNA pools, and demonstrated 99% identity to *Bl. afzelii* strain VS461 and *Bl. tanukii* strain Hk501. However, a GenBank search without restriction to type material indicated that this sequence variant was also identical to several *Bl. afzelii* strains with available complete genome sequences, including *Bl. afzelii* strain PMel (GenBank accession no. CP075447.1).

## 4. Discussion

This study demonstrates that the newly developed *flaB* gene-based profiling approach offers significantly higher performance for the detection of Borreliaceae DNA in ticks compared to conventional nested PCR and V4 *rrs* gene-based profiling. The increased efficiency of the *flaB* gene-based profiling method results from the combination of Borreliaceae-specific PCR primers with NGS technology, which enables the detection of low-abundance targets and co-infecting species within complex DNA mixtures, such as the pools composed of DNA from more than 10 individual tick isolates. The discrepancies for the seven samples detected in this study by the nested PCR assay and V4 *rrs* marker, but not by the *flaB* gene-based profiling, may be attributed to stochastic events, which can occur when trace amounts of the target template are present in the sample [48]. This may have occurred due to the DNA pooling strategy applied, which aimed to maximize the detection of a broad range of Borreliaceae species, potentially at the cost of diluting the pathogen’s DNA in the final sample. Nonetheless, we consider the *rrs* gene-based profiling a valuable complementary tool for the screening of TBPs in studies aimed at detecting a broad range of taxa, despite its lower sensitivity resulting from the use of universal primers.

The *flaB* gene-based profiling method proved not only sensitive, but also informative, at both the species level and the putative strain level. A total of 23 distinct *flaB* sequence variants were identified, corresponding to five recognized Borreliaceae species. The most frequently identified species in this study was *Bl. afzelii*, followed by *Bl. valaisiana*, *Bl. garinii*, *Bl. burgdorferi*, and *B. miyamotoi*. These results are consistent with previous reports on the prevalence of Borreliaceae species, where *Bl. afzelii* is typically the most frequently detected species in both Europe and Poland, usually followed by *Bl. garinii*, *Bl. burgdorferi*, and *Bl. valaisiana* [19,49,50,51]. In a recent study conducted in northern Poland, the two most prevalent Borreliaceae species detected in *I. ricinus* ticks were *Bl. afzelii* (29.4% of infected ticks) and *Bl. garinii* (20.0%), while other species included *Bl. valaisiana* (9.4%), *Bl. burgdorferi* (6.0%), and *B. miyamotoi* (6.3%) [19]. Similarly, a study carried out in the city of Poznan, Poland, revealed a comparable pattern of Borreliaceae species distribution in ticks collected from vegetation, with *Bl. afzelii* and *Bl. garinii* detected in 3.7%, *Bl. valaisiana* in 1.2%, and *B. miyamotoi* in 0.6% of the collected ticks tested [31].

Notably, intra-species sequence diversity was observed, which, in the future, in combination with other markers, may allow for finer differentiation of strain-level identification. For example, *flaB* gene-based profiling enabled the detection of eight distinct *flaB* sequence variants of *Bl. garinii*, whereas previous studies employing dPCR identified only one or two variants when analyzing 116 and 408 ticks, respectively [52,53]. A similar pattern was observed for *Bl. afzelii*, where five *flaB* variants were identified in our study, compared to only one variant detected by dPCR, in which only a subset of samples was sequenced to validate the amplification results [53].

This level of taxonomic resolution was not achieved using the V4 *rrs* gene marker, where lower variability limited species and strain discrimination. For example, *Bl. americana* and *Bl. mayonii* shared an identical V4 *rrs* sequence, underscoring the greater resolving power of the *flaB* gene. *Borrelia americana* has been detected in Poland using the same approach [54]; however, the sequence variants identified in the study were not published, making it impossible to determine whether we detected the same V4 *rrs* sequence variant. Nonetheless, some studies suggest that sequence variation within the *rrs* gene may occur among strains belonging to the same species of Borreliaceae, especially in members of the genus *Borreliella* [31,55].

In our study, it was not possible to establish a direct correspondence between the V4 *rrs* and the *flaB* sequence variants, as the analyses were conducted on pooled DNA samples composed of multiple tick isolates. The presence of mixed Borreliaceae DNA within these pools precludes confident assignment of specific *rrs* and *flaB* variants to the same organism. However, we consider such linkage insufficient for species determination, as any attempt to infer strain-level associations between these markers would be speculative without genomic confirmation. Ultimately, only whole-genome sequencing could provide the resolution needed to determine whether the observed sequence variants represent intra-species diversity (i.e., strains) or distinct species-level lineages.

The *flaB* gene-based profiling approach represents a valuable tool for large-scale screening of tick-borne pathogens, as it enables the detection of mixed Borreliaceae infections and provides more accurate estimates of pathogen prevalence within vector populations. Unlike highly targeted qPCR assays, this method offers broader taxonomic coverage, facilitating the detection of potentially all Borreliaceae species present in a sample through a single assay. Compared to the marker used in the MPAS method [20,27], the sequence fragment employed in the *flaB* gene-based profiling is shorter, making it more cost-effective for NGS. Additionally, it is amplified using degenerate primers, which may potentially allow for the detection of a broader range of strains. At the same time, it remains significantly more cost-effective than metagenomic approaches that rely on sequencing the entire DNA content of a sample, making it particularly suitable for epidemiological surveillance and high-throughput environmental monitoring.

## 5. Conclusions

This study demonstrates that the *flaB* gene remains one of the most effective molecular markers for the detection of Borreliaceae DNA in *I. ricinus* ticks. The newly developed *flaB* gene-based profiling approach significantly improved analytical efficiency and throughput by enabling the detection of multiple species in pooled DNA samples derived from more than 10 individual ticks. In addition to detecting five Borreliaceae species, this method also allowed for putative strain-level resolution, offering deeper insight into the genetic diversity of *Borrelia* and *Borreliella* populations in the studied environment. Moreover, our assay is compatible with any NGS platform, as its performance is based on the abi-flaB primer sequences. Depending on the workflow, the primers can be extended at the 5′ ends with indexes and platform-specific adapters, or with adapters that enable the conversion of the library for use with any NGS system. Compared to conventional nested PCR and V4 *rrs* gene-based profiling, the new approach provided a broader and more detailed picture of Borreliaceae composition. As such, it represents a valuable molecular tool for future diagnostic applications, ecological surveys, and long-term monitoring of tick-borne pathogens.

## Figures and Tables

**Table 1:** The results of McNemar’s exact test comparing the performance of the three Borreliaceae DNA detection methods.

Compared Methods	McNemar Test Statistic ^1^	*p*-Value	Significant Difference ^2^
*flaB* profiling vs. nested PCR	4	1.09 × 10^−5^	Yes
*flaB* profiling vs. V4 *rrs* profiling	5	7.43 × 10^−6^	Yes
V4 *rrs* profiling vs. nested PCR	16	0.8642	No

^1^ Testing performed using exact McNemar’s test for paired nominal data. ^2^ Significance threshold: *p* < 0.05

## Data Availability

The raw *flaB* gene insert sequences are available in NCBI under BioProject accession number PRJNA1259556. The ASV sequences obtained in this study have been published in GenBank under the accession numbers PQ727213, PQ727214, PQ727215-PQ727236 (*flaB*), and PV151552-PV151564 (V4 *rrs*).

## Supplementary Materials

The following supporting information can be downloaded at https://www.mdpi.com/article/10.3390/pathogens14050506/s1, Table S1: Borreliaceae *flaB* sequences used for developing abi-flaB primer set; Table S2: The *flaB* sequences used in phylogentic analysis. Sequences generated in this study are in bold (GenBank acc. nos. PQ727213-PQ727236); Table S3: Comparison of the detected presence (+) and absence (−) of Borreliaceae DNA in DNA pools; Table S4: The detection (+) and absence (−) of Borreliaceae species (*Bl. afzelii*, *Bl. valaisiana*, *Bl. garinii*, *Bl. burgdorferi* and *B. miyamotoi*) for *flaB* gene- and V4 *rrs* gene-based profiling; Figure S1: The results of the nested PCR test based on the amplification of a 604 bp fragment of the *flaB* gene; Figure S2: Amino acid sequences of the *flaB* gene sequence variants found in this study.

## References

[1] Kulisz J., Hoeks S., Kunc-Kozioł R., Woźniak A., Zając Z., Schipper A.M., Cabezas-Cruz A., Huijbregts M.A.J. (2024). Spatiotemporal trends and covariates of Lyme borreliosis incidence in Poland, 2010–2019. Sci. Rep..

[2] Medlock J.M., Hansford K.M., Bormane A., Derdakova M., Estrada-Peña A., George J.-C., Golovljova I., Jaenson T.G.T., Jensen J.-K., Jensen P.M. (2013). Driving forces for changes in geographical distribution of *Ixodes ricinus* ticks in Europe. Parasites Vectors.

[3] De La Fuente J., Estrada-Peña A., Rafael M., Almazán C., Bermúdez S., Abdelbaset A.E., Kasaija P.D., Kabi F., Akande F.A., Ajagbe D.O. (2023). Perception of Ticks and Tick-Borne Diseases Worldwide. Pathogens.

[4] Le Dortz L.L., Rouxel C., Polack B., Boulouis H.-J., Lagrée A.-C., Deshuillers P.L., Haddad N. (2024). Tick-borne diseases in Europe: Current prevention, control tools and the promise of aptamers. Vet. Parasitol..

[5] Pustijanac E., Buršić M., Millotti G., Paliaga P., Iveša N., Cvek M. (2024). Tick-Borne Bacterial Diseases in Europe: Threats to public health. Eur. J. Clin. Microbiol. Infect. Dis..

[6] Burn L., Tran T.M.P., Pilz A., Vyse A., Fletcher M.A., Angulo F.J., Gessner B.D., Moïsi J.C., Jodar L., Stark J.H. (2023). Incidence of Lyme Borreliosis in Europe from National Surveillance Systems (2005–2020). Vector-Borne Zoonotic Dis..

[7] Paradowska-Stankiewicz I., Zbrzeźniak J., Skufca J., Nagarajan A., Ochocka P., Pilz A., Vyse A., Begier E., Dzingina M., Blum M. (2023). A Retrospective Database Study of Lyme Borreliosis Incidence in Poland from 2015 to 2019: A Public Health Concern. Vector-Borne Zoonotic Dis..

[8] Barbour A.G., Gupta R.S. (2021). The Family *Borreliaceae* (Spirochaetales), a Diverse Group in Two Genera of Tick-Borne Spirochetes of Mammals, Birds, and Reptiles. J. Med. Entomol..

[9] Gupta R.S. (2019). Distinction between *Borrelia* and *Borreliella* is more robustly supported by molecular and phenotypic characteristics than all other neighbouring prokaryotic genera: Response to Margos’ et al. “The genus *Borrelia* reloaded” (PLoS ONE 13(12): e0208432). PLoS ONE.

[10] Estrada-Peña A., Cutler S., Potkonjak A., Vassier-Tussaut M., Van Bortel W., Zeller H., Fernández-Ruiz N., Mihalca A.D. (2018). An updated meta-analysis of the distribution and prevalence of *Borrelia burgdorferi* s.l. in ticks in Europe. Int. J. Health Geogr..

[11] Dong Y., Zhou G., Cao W., Xu X., Zhang Y., Ji Z., Yang J., Chen J., Liu M., Fan Y. (2022). Global seroprevalence and sociodemographic characteristics of *Borrelia burgdorferi* sensu lato in human populations: A systematic review and meta-analysis. BMJ Glob. Health.

[12] Tokarz R., Lipkin W.I. (2021). Discovery and Surveillance of Tick-Borne Pathogens. J. Med. Entomol..

[13] Zinck C.B., Raveendram Thampy P., Uhlemann E.M.E., Adam H., Wachter J., Suchan D., Cameron A.D.S., Rego R.O.M., Brisson D., Bouchard C. (2023). Variation among strains of *Borrelia burgdorferi* in host tissue abundance and lifetime transmission determine the population strain structure in nature. PLoS Pathog..

[14] Carter C.J., Bergström S., Norris S.J., Barbour A.G. (1994). A family of surface-exposed proteins of 20 kilodaltons in the genus Borrelia. Infect. Immun..

[15] Stone B.L., Brissette C.A. (2017). Host Immune Evasion by Lyme and Relapsing Fever Borreliae: Findings to Lead Future Studies for Borrelia miyamotoi. Front. Immunol..

[16] Wodecka B., Leońska A., Skotarczak B. (2010). A comparative analysis of molecular markers for the detection and identification of Borrelia spirochaetes in *Ixodes ricinus*. J. Med. Microbiol..

[17] Graham C.B., Maes S.E., Hojgaard A., Fleshman A.C., Sheldon S.W., Eisen R.J. (2018). A molecular algorithm to detect and differentiate human pathogens infecting *Ixodes scapularis* and *Ixodes pacificus* (Acari: Ixodidae). Ticks Tick-Borne Dis..

[18] Wodecka B. (2011). *flaB* Gene as a Molecular Marker for Distinct Identification of Borrelia Species in Environmental Samples by the PCR-Restriction Fragment Length Polymorphism Method. Appl. Environ. Microbiol..

[19] Wodecka B., Kolomiiets V. (2023). Genetic Diversity of *Borreliaceae* Species Detected in Natural Populations of *Ixodes ricinus* Ticks in Northern Poland. Life.

[20] Osikowicz L.M., Rizzo M.R., Hojgaard A., Maes S.E., Eisen R.J. (2024). Detection of *Borrelia burgdorferi* sensu lato species in host-seeking *Ixodes* species ticks in the United States. Ticks Tick-Borne Dis..

[21] Yang J., Liu Z., Guan G., Che R., Niu Q., Li Y., Liu J., Ma M., Ren Q., Liu A. (2012). Evaluation of molecular methods for detection of *Borrelia burgdorferi* senso lato in ticks. Diagn. Microbiol. Infect. Dis..

[22] Zhai B., Niu Q., Liu Z., Yang J., Pan Y., Li Y., Zhao H., Luo J., Yin H. (2018). First detection and molecular identification of Borrelia species in Bactrian camel (*Camelus bactrianus*) from Northwest China. Infect. Genet. Evol..

[23] Binetruy F., Garnier S., Boulanger N., Talagrand-Reboul É., Loire E., Faivre B., Noël V., Buysse M., Duron O. (2020). A novel Borrelia species, intermediate between Lyme disease and relapsing fever groups, in neotropical passerine-associated ticks. Sci. Rep..

[24] Wodecka B., Michalik J., Grochowalska R. (2022). Red Foxes (*Vulpes vulpes*) Are Exposed to High Diversity of *Borrelia burgdorferi* Sensu Lato Species Infecting Fox-Derived *Ixodes* Ticks in West-Central Poland. Pathogens.

[25] Trzebny A., Slodkowicz-Kowalska A., Becnel J.J., Sanscrainte N., Dabert M. (2020). A new method of metabarcoding Microsporidia and their hosts reveals high levels of microsporidian infections in mosquitoes (Culicidae). Mol. Ecol. Resour..

[26] Michelet L., Delannoy S., Devillers E., Umhang G., Aspan A., Juremalm M., Chirico J., van der Wal F.J., Sprong H., Boye Pihl T.P. (2014). High-throughput screening of tick-borne pathogens in Europe. Front. Cell. Infect. Microbiol..

[27] Hojgaard A., Osikowicz L.M., Eisen L., Eisen R.J. (2020). Evaluation of a novel multiplex PCR amplicon sequencing assay for detection of human pathogens in *Ixodes* ticks. Ticks Tick. Borne Dis..

[28] Siuda K. (1993). Kleszcze Polski (Acari: Ixodida).: Systematyka i Rozmieszczenie.

[29] Rijpkema S., Golubic D., Molkenboer M., Verbeek-De Kruif N., Schellekens J. (1996). Identification of four genomic groups of *Borrelia burgdorferi* sensu lato in *Ixodes ricinus* ticks collected in a Lyme borreliosis endemic region in northern Croatia. Exp. Appl. Acarol..

[30] Michalik J., Wodecka B., Liberska J., Dabert M., Postawa T., Piksa K., Stańczak J. (2020). Diversity of *Borrelia burgdorferi* sensu lato species in *Ixodes* ticks (Acari: Ixodidae) associated with cave-dwelling bats from Poland and Romania. Ticks Tick-Borne Dis..

[31] Liberska J., Michalik J.F., Olechnowicz J., Dabert M. (2024). Co-Occurrence of *Borrelia burgdorferi* Sensu Lato and Babesia spp. DNA in *Ixodes ricinus* Ticks Collected from Vegetation and Pets in the City of Poznań, Poland. Pathogens.

[32] Hall T.A. (1999). BioEdit: A user-friendly biological sequence alignment editor and analysis program for Windows95/98/NT. Nucleic Acids Symp. Ser..

[33] Makowska N., Philips A., Dabert M., Nowis K., Trzebny A., Koczura R., Mokracka J. (2020). Metagenomic analysis of β-lactamase and carbapenemase genes in the wastewater resistome. Water Res..

[34] Therese K.L., Anand A.R., Madhavan H.N. (1998). Polymerase chain reaction in the diagnosis of bacterial endophthalmitis. Br. J. Ophthalmol..

[35] Hannon G.J. (2010). FASTX-Toolkit. https://bio.tools/fastx-toolkit.

[36] Bolyen E., Rideout J.R., Dillon M.R., Bokulich N.A., Abnet C.C., Al-Ghalith G.A., Alexander H., Alm E.J., Arumugam M., Asnicar F. (2019). Reproducible, interactive, scalable and extensible microbiome data science using QIIME 2. Nat. Biotechnol..

[37] Callahan B.J., McMurdie P.J., Rosen M.J., Han A.W., Johnson A.J.A., Holmes S.P. (2016). DADA2: High-resolution sample inference from Illumina amplicon data. Nat. Methods.

[38] Edgar R.C. (2018). UNCROSS2: Identification of cross-talk in 16S rRNA OTU tables. BioRxiv.

[39] Glöckner F.O., Yilmaz P., Quast C., Gerken J., Beccati A., Ciuprina A., Bruns G., Yarza P., Peplies J., Westram R. (2017). 25 years of serving the community with ribosomal RNA gene reference databases and tools. J. Biotechnol..

[40] Quast C., Pruesse E., Yilmaz P., Gerken J., Schweer T., Yarza P., Peplies J., Glöckner F.O. (2012). The SILVA ribosomal RNA gene database project: Improved data processing and web-based tools. Nucleic Acids Res..

[41] Yilmaz P., Parfrey L.W., Yarza P., Gerken J., Pruesse E., Quast C., Schweer T., Peplies J., Ludwig W., Glöckner F.O. (2014). The SILVA and “All-species Living Tree Project (LTP)” taxonomic frameworks. Nucl. Acids Res..

[42] Zhang Z., Schwartz S., Wagner L., Miller W. (2000). A Greedy Algorithm for Aligning DNA Sequences. J. Comput. Biol..

[43] Morgulis A., Coulouris G., Raytselis Y., Madden T.L., Agarwala R., Schäffer A.A. (2008). Database indexing for production MegaBLAST searches. Bioinformatics.

[44] Nicholas K.B., Nicholas H.B. GeneDoc: A Tool for Editing and Annotating Multiple Sequence Alignments; 1997. https://api.semanticscholar.org/CorpusID:81058551.

[45] McNemar Q. (1947). Note on the sampling error of the difference between correlated proportions or percentages. Psychometrika.

[46] Shimodaira H., Hasegawa M. (1999). Multiple Comparisons of Log-Likelihoods with Applications to Phylogenetic Inference. Mol. Biol. Evol..

[47] Kumar S., Stecher G., Tamura K. (2016). MEGA7: Molecular Evolutionary Genetics Analysis Version 7.0 for Bigger Datasets. Mol. Biol. Evol..

[48] Taberlet P., Griffin S., Goossens B., Questiau S., Manceau V., Escaravage N., Waits L.P., Bouvet J. (1996). Reliable genotyping of samples with very low DNA quantities using PCR. Nucleic Acids Res..

[49] Liberska J.A., Michalik J.F., Dabert M. (2023). Exposure of dogs and cats to Borrelia miyamotoi infected *Ixodes ricinus* ticks in urban areas of the city of Poznań, west-central Poland. Ticks Tick-Borne Dis..

[50] Strnad M., Hönig V., Růžek D., Grubhoffer L., Rego R.O.M. (2017). Europe-Wide Meta-Analysis of *Borrelia burgdorferi* Sensu Lato Prevalence in Questing *Ixodes ricinus* Ticks. Appl. Environ. Microbiol..

[51] Sawczyn-Domańska A., Zwoliński J., Kloc A., Wójcik-Fatla A. (2023). Prevalence of *Borrelia*, *Neoehrlichia mikurensis* and *Babesia* in ticks collected from vegetation in eastern Poland. Exp. Appl. Acarol..

[52] Kulisz J., Zając Z., Foucault-Simonin A., Woźniak A., Filipiuk M., Kloskowski J., Rudolf R., Corduneanu A., Bartosik K., Moutailler S. (2024). Wide spectrum of tick-borne pathogens in juvenile *Ixodes ricinus* collected from autumn-migrating birds in the Vistula River Valley, Poland. BMC Vet. Res..

[53] Zając Z., Kulisz J., Woźniak A., Bartosik K., Foucault-Simonin A., Moutailler S., Cabezas-Cruz A. (2023). Tick Activity, Host Range, and Tick-Borne Pathogen Prevalence in Mountain Habitats of the Western Carpathians, Poland. Pathogens.

[54] Dunaj J., Drewnowska J., Moniuszko-Malinowska A., Swiecicka I., Pancewicz S. (2021). First metagenomic report of *Borrelia americana* and *Borrelia carolinensis* in Poland—A preliminary study. Ann. Agric. Environ. Med..

[55] Lau A.C.C., Qiu Y., Moustafa M.A.M., Nakao R., Shimozuru M., Onuma M., Mohd-Azlan J., Tsubota T. (2020). Detection of *Borrelia burgdorferi* sensu lato and relapsing fever borrelia in feeding *Ixodes* ticks and rodents in Sarawak, Malaysia: New geographical records of borrelia yangtzensis and borrelia miyamotoi. Pathogens.

